# Impact of PGT Introduction on IVF Laboratory Workload: Lessons Learned from a Single-Center Experience of 5258 Biopsies over a 10-Year Period

**DOI:** 10.3390/life15091351

**Published:** 2025-08-26

**Authors:** Stefano Canosa, Luisa Delle Piane, Danilo Cimadomo, Alberto Revelli, Gianluca Gennarelli, Daniela Guidetti, Cristina Garello, Francesca Granella, Francesca Evangelista, Giuseppe Monelli, Lucia Clemente, Antonio Capalbo, Laura Rienzi, Ugo Sorrentino, Daniela Zuccarello, Francesca Bongioanni

**Affiliations:** 1 IVIRMA Global Research Alliance, Livet, Via Tiziano Vecellio 3, 10126 Turin, Italy; l.dellepiane@iol.it (L.D.P.); aerre99@yahoo.com (A.R.); gennarelligl@gmail.com (G.G.); daniela.guidetti@tin.it (D.G.); cristina.garello@generapma.it (C.G.); francesca.granella@generapma.it (F.G.); francesca.evangelista@generapma.it (F.E.); giuseppe.monelli@generapma.it (G.M.); lucia.clemente@generapma.it (L.C.); francesca.bongioanni@generapma.it (F.B.); 2 IVIRMA Global Research Alliance, Genera, Clinica Valle Giulia, 00197 Rome, Italy; danilo.cimadomo@ivirma.com (D.C.); laura.rienzi@ivirma.com (L.R.); 3 Department of Biology and Biotechnology “Lazzaro Spallanzani”, University of Pavia, 27100 Pavia, Italy; 4 Juno Genetics, 00100 Rome, Italy; antonio.capalbo@junogenetics.com; 5 Unit of Molecular Genetics, Center for Advanced Studies and Technology (CAST), ‘G. D’Annunzio’ University of Chieti-Pescara, 66100 Chieti, Italy; 6 Department of Biomolecular Sciences, University of Urbino “Carlo Bo”, 61029 Urbino, Italy; 7 Department of Women’s and Children’s Health, University Hospital of Padova, 35128 Padova, Italy; ugo.sorrentino@unipd.it; 8 Unit of Medical Genetics and Genomics, San Bortolo Hospital, ULSS n.8 “Berica”, 36100 Vicenza, Italy; dottoressazuccarello@gmail.com

**Keywords:** IVF, PGT, blastocyst biopsy, lab workload, laboratory management

## Abstract

The aim of our study was to provide a retrospective single-center experience of the additional workload associated with routine PGT, including embryologist training and suggested staffing levels. A total of 4945 IVF cycles were retrospectively considered, of which 1680 were PGT cycles with a total of 5258 biopsied blastocysts. An exponential increase in the proportion of PGTs over OPUs was observed, from 0.2% in 2015 to 72.9% in 2024. The number of viable embryos for biopsy was significantly increased by the systematic adoption of an extended embryo culture and the concomitant transition from a day 2 Double Embryo Transfer (DET) to a day 5 Single Blastocyst Transfer (SET) policy in 2020. In order to cope with the increasing workload, a concomitant increase in the number of embryologists involved in blastocyst biopsy was adopted, with a second embryologist in 2020, a third in 2021, and a fourth in 2022, with a trend comparable to that observed for the proportion of PGT cycles over IVF cycles performed during the study period. The appropriate number of staff required for the IVF laboratory was calculated using the Staffing Model for ART (smART) calculator, based on 12 routine IVF procedures. An optimal balance between operational procedures and staffing levels was achieved when the difference (Δ) was ≤10%, ensuring the efficient maintenance of PGT in the IVF laboratory.

## 1. Introduction

Preimplantation Genetic Testing (PGT) involves embryo biopsy coupled with Comprehensive chromosomal testing (CCT) to analyze DNA from embryos at the cleavage or blastocyst stage for the assessment of genetic abnormalities or for HLA typing [1]. In recent years, several pieces of biological and clinical evidence have been reported on the high efficacy of trophectoderm (TE) biopsy over blastomere or polar body biopsy to improve embryo (de)selection, establishing it as the most widely used strategy worldwide [2,3,4,5]. PGT comprises three subcategories: (i) PGT for monogenic or single gene disorders (PGT-M, formerly known as Preimplantation Genetic Diagnosis, PGD), performed to reduce the risk of couples affected by or carrying a genetic disease having a child with the same genetic disorder [6,7]; (ii) PGT for aneuploidy (PGT-A, formerly known as Preimplantation Genetic Screening, PGS), used to detect aneuploidies arising from both male and female meiosis in in vitro generated embryos, with the ultimate aim of minimizing the risk of adverse clinical outcomes (i.e., implantation failure, miscarriage, and live births affected by chromosomal abnormalities) by excluding from transfer embryos carrying numerical changes in chromosomes [8,9,10,11,12]; and (iii) PGT for structural chromosomal rearrangements (PGT-SR) offered in cases of balanced structural chromosomal rearrangements (such as translocations or inversions) to prevent the risk of having a pregnancy or child with an unbalanced structural abnormality, which involves extra or missing genetic material and typically results in pregnancy loss [8,13]. The use of PGT has evolved in recent years, including improvements in embryo culture, biopsy, transfer, and the accuracy of genetic testing. Interestingly, with appropriate training and working in a standardized in vitro fertilization (IVF) environment, very high consistency and reproducibility of CCT on blastocyst biopsy has been observed between different practitioners [14]. Besides the recommendation that the same trained embryologist perform both the TE biopsy and the tubing, a second operator available for manual witness is necessary to ensure traceability. Nevertheless, this ideal scenario with two trained embryologists at a time can greatly affect the clinical schedule of the IVF lab [15,16]. We retrospectively collected the number of PGT cycles performed in our clinic since its introduction in 2015 and the training data of each embryologist involved in TE biopsy and calculated the ideal number of staff required to perform the laboratory procedures. In order to advise IVF laboratories planning to introduce PGT into their workflow, the aim of the current study was to provide a single-center experience of how to efficiently manage the impact of the additional workload required when PGT is routinely performed, while maintaining adequate quality control.

## 2. Materials and Methods

### 2.1. Study Design

This is a single-center, observational, retrospective study to assess the impact of PGT-related procedures and protocols on the daily workload of an IVF laboratory. The study was conducted in accordance with the Declaration of Helsinki, and ethics committee approval was obtained for the retrospective analysis of pseudonymized data by the Institutional Review Board of Citta’ Della Salute e Della Scienza di Torino. We included all patients who underwent IVF treatment at our private clinic between January 2015 and December 2024. During this period, 4945 IVF cycles were performed, of which 1680 were PGT cycles with a total of 5258 biopsied blastocysts. PGT has been used to test for monogenic variants (PGT-M), aneuploidy (PGT-A), or large structural variants (PGT-SR). PGT-M was offered to patients carrying high-penetrance pathogenic variants who are at risk of having affected children. In this case, a preclinical setup was carried out consisting of the collection of blood sample to perform genome-wide haplotyping using the parents of the affected individual as a phasing reference [6]. In these patients, the euploidy test was carried out on healthy or carrier blastocysts until September 2023 and on all available blastocysts thereafter. The success rates and risks of error of the procedure were then explained according to the specific genetic disease for which the couple is at increased risk. Carrier Genetic Testing (CGT) for the most common monogenic diseases was performed only at the patient’s request. In the case of PGT-SR, a karyotype was taken from both partners, and a feasibility analysis was carried out based on the technical method used by the PGT laboratory [8].

### 2.2. IVF and Biopsy Procedures

During the study period, ovarian stimulation was performed according to patients’ ovarian reserve markers (Antral Follicle Count, AFC, and Anti-Mullerian Hormone, AMH) and expected response to gonadotropin stimulation. Insemination was performed by Intracytoplasmic Sperm Injection (ICSI) within 4 h of oocyte collection. After ICSI, the oocytes were transferred to cleavage medium (Cleavage, Origio, Ballerup, Ireland) overlaid with mineral oil (LifeGuard Oil, LifeGlobal IVF, Trumbull, CT, USA), and normal fertilization was confirmed by the presence of two pronuclei (2PN) and extrusion of the second polar body 16–18 h later. Zygotes were cultured in cleavage medium until day 3 of development, at which time the medium was changed to a stage-specific medium (Sequential Blast, Origio, Ballerup, Ireland) until the fully expanded blastocyst stage (days 5–7). Embryo culture was performed in a controlled humidified atmosphere (37 °C, 6% CO_2_) at low oxygen tension (5%) in BT37 (Planer, Origio, Ballerup, Ireland) benchtop incubators. TE biopsy was performed on expanded blastocysts when the Inner Cell Mass (ICM) was clearly visible, with or without herniated cells, using simultaneous laser-assisted zona opening and sequential retrieval of trophectoderm fragments. Six to eight TE cells were retrieved, washed in buffered medium, and collected in small reaction tubes for amplification-based testing (tubing procedure). All daily biopsies were performed by the same operator in a single slot in the morning before 1 pm. Blastocyst morphology was assessed immediately prior to TE biopsy according to Gardner score and reviewed in real time by two senior embryologists (with ≥6 years of experience in IVF) for verification and consistency. Manual witnessing was performed by a second operator at the following stages, as previously described [17]: (i) before and after the biopsy, to confirm that the embryo and sample number matched; (ii) during tubing, to confirm that the sample labelling matched the label on the corresponding tube; (iii) during vitrification, when the blastocyst was placed in the vitrification well and labelled; (iv) when the genetic results were received, to ensure correspondence between embryo identification and euploidy status; (v) at the time of embryo selection for transfer and at the time of the warming procedure. Vitrification was performed within 30 min from trophectoderm biopsy on collapsed blastocysts with Cryotop carrier using the Vitrification Kit (Kitazato, Shizuoka, Japan). Comprehensive chromosomal testing was performed by Next-Generation Sequencing (NGS) at an external lab. We did not report “embryos with a PGT-A result in the mosaic range” [18]. Re-biopsy was considered if the genetic diagnosis was inconclusive, with a benchmark value ≤ 5% [19].

### 2.3. Training for Biopsy and Tubing

Embryo biopsy and tubing training were carried out in accordance with the ESHRE PGT Good Practice Recommendations [19] and were supervised by an operator with recognized expertise in PGT (≥3 years of experience in biopsy at the time of training) [20]. In particular, training in biopsy consisted of two phases: preclinical training and supervised clinical training. Pre-clinical training used at least 50 degenerate embryos to practice all steps (i.e., opening the zona pellucida, cell collection, washing and tubing) of the biopsy procedure. Efficient transfer of biopsied cells into reaction tubes was considered a critical step for the success of the PGT cycle. Accurate sample handling was confirmed by the witness operator to avoid exogenous DNA contamination. Supervised clinical training included at least 20 additional blastocyst biopsies. To evaluate clinical training, post-biopsy damage and survival after continued culture were monitored. In addition, biopsy sample damage/lysis and amplification results were assessed.

### 2.4. Statistical Analysis

The primary aim of this study was to describe our clinic’s experience of the evolution of the IVF laboratory workload since PGT was introduced in 2015 and how this has been managed over time. The number of oocyte retrievals, PGT cycles, and the number of blastocysts undergoing biopsy and vitrification were extracted from our medical records software. The number of embryologists involved in the PGT procedures and their years of experience in IVF and biopsy were reported. The number of laser shots used and the timings (minutes) per biopsied blastocyst were also recorded by a second operator in charge of the manual witnessing using a timer: time to TE biopsy (calculated from the first laser shot to open the ZP to the release of the biopsied fragment into the culture medium) and time to cell tubing (calculated from the identification of the tube to the release of the biopsied fragment into the tube itself). The inter-operator variability was assessed using the Kruskal–Wallis test. The appropriate number of staff required for the IVF laboratory was calculated using the Staffing Model for ART (smART) calculator [21]. Analyses were performed using GraphPad Prism V7 software. Continuous variables were expressed as mean ± standard deviation (SD), while categorical variables were expressed as absolute values and percentages.

## 3. Results

### 3.1. Workload Analysis of the Embryo Biopsies Performed

During the study period, a total of 4945 IVF cycles were performed, of which 1680 were PGT cycles. As shown in Table 1, we observed an exponential increase in the number of PGT cycles performed over time, particularly between 2019 and 2022. The proportion of PGT cycles compared to oocyte retrievals increased with the same trend, ranging from 0.2% in 2015 to 72.9% in 2024. In particular, 95.3% (N = 1602/1680) of cycles were requested for PGT-A, 3.3% (N = 55/1680) for PGT-M, and 1.4% (N = 23/1680) for PGT-SR.

**Figure 1:**
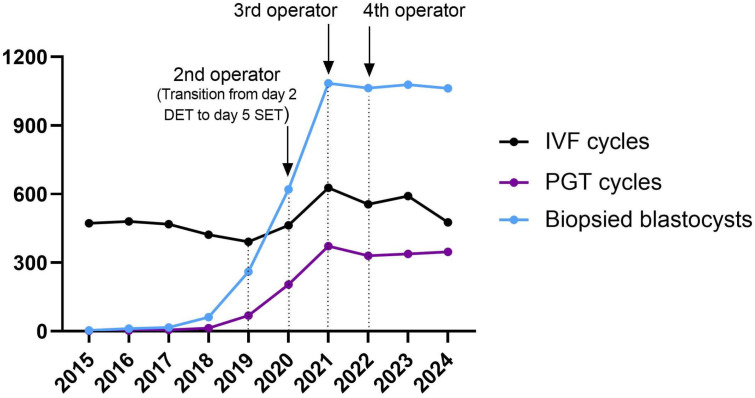
Trend of biopsies (light blue line), PGT (dark purple line), and IVF cycles (black line) performed during the study period.

Specifically, in order to deal with the exponential increase in the workload of the IVF laboratory, an implementation in the training program was adopted, increasing the number of embryologists involved in blastocyst biopsy, with the addition of a second embryologist in 2020, a third in 2021, and a fourth in 2022 (Figure 1).

Overall, an average of 3.1 (5258/1680) blastocysts were biopsied per cycle, ranging from 0 to 16 blastocysts biopsied per patient, with a peak of 22 blastocysts biopsied in a single working day in 2023 (with 4 operators trained for PGT). As expected, the total number of days per year that biopsies were performed gradually increased, reaching a maximum of 247 days (67.7%, 247/365) in 2023 (Table 2).

**Figure 2:**
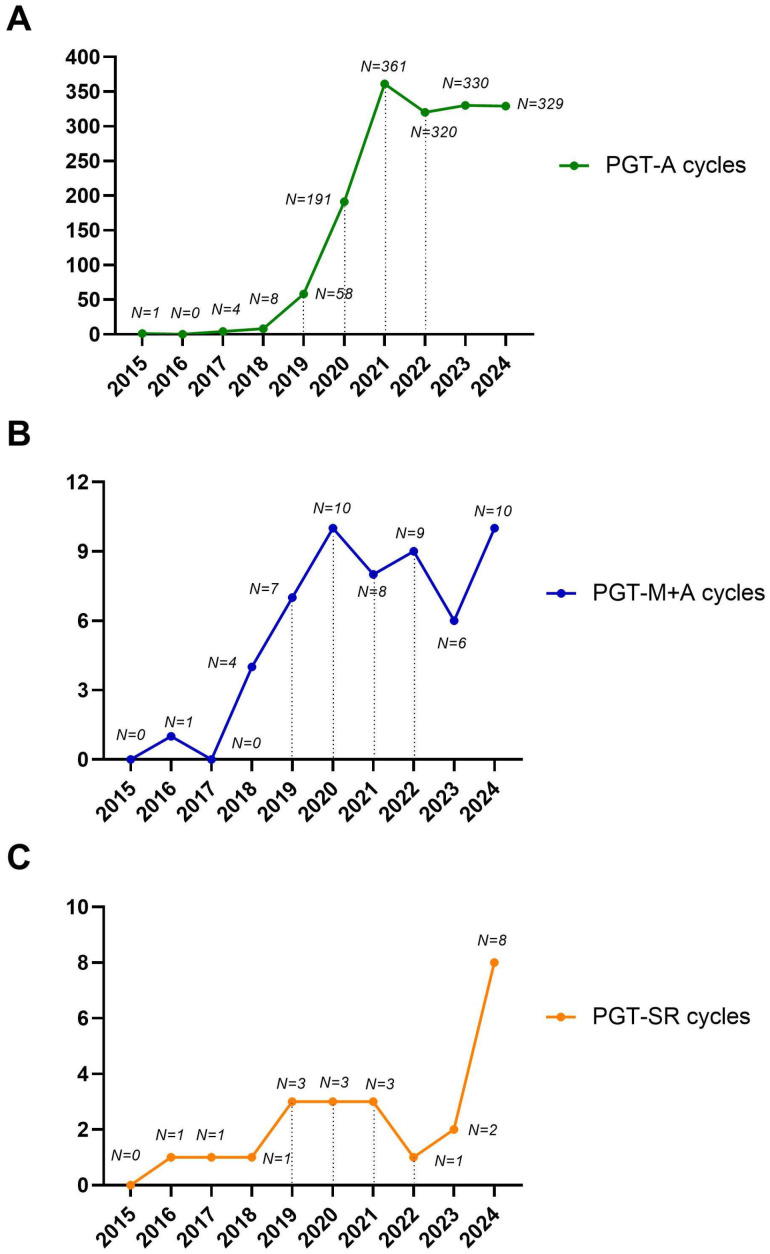
Detailed trends for PGT-A (**A**), PGT-M+A (**B**), and PGT-SR (**C**) over the study period. The number of cycles performed per year is shown.

As expected, PGT-A was the driving force behind the observed exponential increase in biopsies during the study period. Conversely, we observed a moderate increase for PGT-M and a stable trend for PGT-SR over the study period (Figure 2A–C). The increase in PGT-M and SR observed during the study period was not due to a higher prevalence of these patients but rather to the fact that prior to 2015, these patients were not treated at our center, as PGT was not available. Instead, they were referred to other private centers in Italy.

### 3.2. Timing Analysis of Embryo Biopsy Procedures

The four operators progressively introduced into the daily PGT practice for biopsy and tubing procedures had 17.5 ± 8.6 years of experience in IVF (range 8–27 years) and 4.5 ± 3.1 years of experience in biopsy (range 2–9 years). The mean number of laser shots used to open the zona pellucida was 7.8 ± 2.5 per biopsy (range 3–13 shots), the mean time to perform TE biopsy was 1.5 ± 0.6 min (range 1–3 min), and the mean time to perform cell tubing was 1.2 ± 0.2 min (range 0.9–2 min). Of the 5258 blastocysts tested, 117 (2.2%) required a re-biopsy after being reported with an inconclusive result. The variability between the four operators is described in Table 3. No significant differences were observed in the number of laser shots (*p* = 0.32), time for biopsy (*p* = 0.49), time for tubing (*p* = 0.72), and the number of non-conclusive results (*p* = 0.68).

### 3.3. Suggested Laboratory Staffing Calculation

The Staffing Model for ART (smART) was used to calculate the suggested staffing levels for the study period based on the activity of our center [21]. Table 4 shows the number of operators involved in PGT-related procedures, the total number of embryologists in the laboratory, the suggested number identified by the calculator, and the differences (Δ) within the staffing level. Between 2015 and 2019, only 1 out of 4 embryologists (25%) was involved in PGT-related procedures. From 2020, a fifth embryologist was recruited, and a second was trained in PGT, increasing the ratio to 2:5 (40%). This ratio increased to 3:6 (50%) in 2021 and to 4:6 (67%) in 2022, 2023, and 2024. Our aim would be to further increase the ratio to 5:6 (83%) in 2025, as a fifth embryologist is currently under training. On average, the total number of IVF operators was one less than the number suggested by the calculator over the study period.

**Figure 3:**
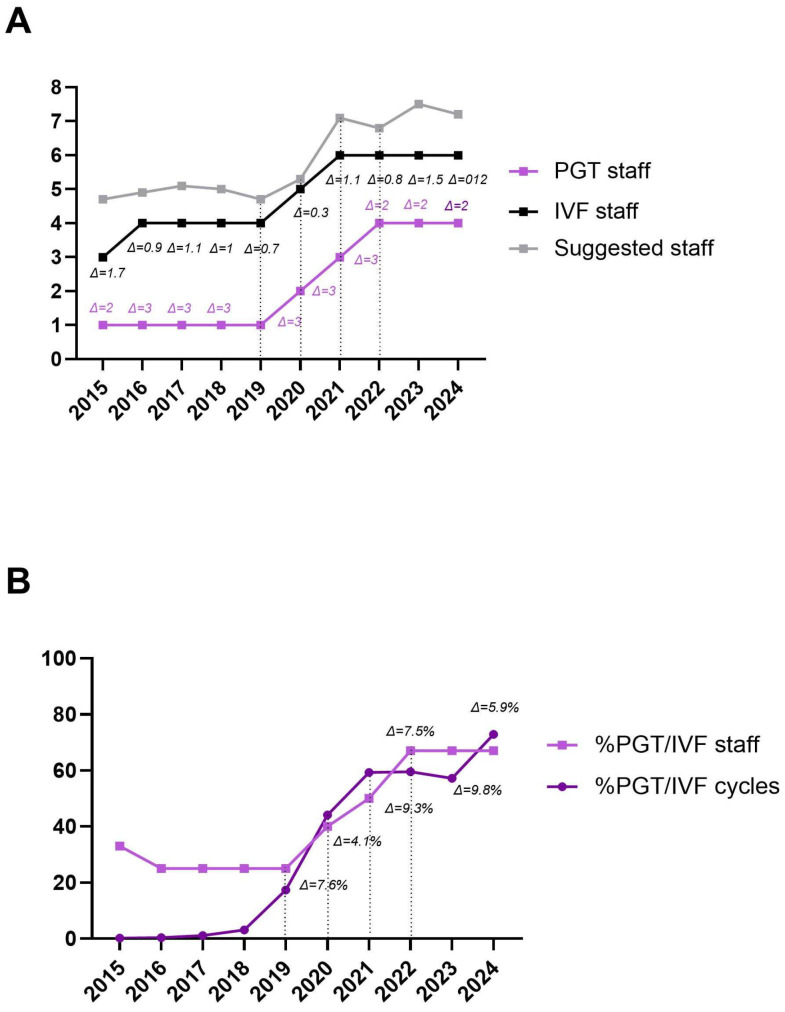
Trend of the number of operators involved in PGT-related procedures (light purple line) and the number of operators in the IVF laboratory (black line), in relation to the suggested number identified by the smART calculator (grey line). The differences (Δ) between PGT and IVF operators (light purple text) and between IVF and suggested operators (black text) are shown (**A**). Trend of the proportion of PGT operators among IVF operators (light purple line, squared dots) compared to the proportion of PGT cycles among IVF cycles performed (dark purple line, rounded dots). Differences (Δ) from 2019 onwards are shown (**B**).

A similar trend was observed when comparing the number of operators involved in PGT procedures to the total number of embryologists, as well as the proportion of PGT cycles to the total IVF cycles during the study period. This suggests that an optimal balance between operational procedures and staffing levels occurs when the difference (Δ) is ≤10% (Figure 3A,B).

## 4. Discussion

According to the latest evidence, blastocyst culture combined with CCT and euploid embryo transfer is the most efficient protocol to increase IVF outcomes [22,23,24]. The exponential increase in PGT cycles observed in our center from 0.2% in 2015 to 72.9% in 2024 reflects the growing clinical confidence in PGT, increased demand from patients (especially older women), and improved technology and operational processes (e.g., blastocyst biopsy and NGS technology). According to the latest available ESHRE PGT Consortium reports, up to 2018, PGT (PGT-M/SR/A combined) accounted for 7.1% of all IVF cycles [25,26,27]. A similar trend was observed across 27 countries in 2019 [28]. In contrast, in the United States, the proportion of IVF cycles involving PGT increased from 21% in 2015 to 44.9% in 2018 [29,30]. Based on these data, the PGT adoption rate at our center was extremely high. In particular, it can be explained by the following reasons: (i) improved genetic counselling was gradually introduced for all patients with the aim of discussing, based on the woman’s age and clinical history, the expected risk of embryonic aneuploidies or transmission of genetic mutations/chromosomal rearrangements [31]; (ii) a DuoStim protocol was suggested for poor prognosis patients with reduced ovarian reserve, emphasizing the importance of obtaining a higher number of oocytes and blastocysts to increase the chances of having at least one euploid/healthy blastocyst available for transfer [32]; (iii) the systematic adoption of an extended embryo culture coupled with the transition from a day 2 DET to a day 5 SET policy [33]. Patients choosing PGT-A were mainly referred to this technique with the following indications: advanced maternal age alone (18.4%, N = 300/1627), recurrent pregnancy loss or repeated implantation failure alone (1.2%, N = 20/1627), their combinations (72.6%, N = 1181/1627), or the couple’s will (7.8%, N = 126/1627). Advanced maternal age (AMA, ≥35 years) represents the most common cause of infertility and the major factor of aneuploidy, and multiple studies have shown that early spontaneous abortion is also significantly related to the incidence of aneuploidy [34]. The mean age of our population was quite high (38.7 ± 3.4 years, range 29–48), which is consistent with the fact that the main reason for the growing number of couples requiring IVF and PGT in Italy is the increasing mean maternal age.

On the other hand, each step of the PGT framework requires an experienced operator. Although international recommendations on the topic are still scarce, these procedural parameters should be considered as essential skills for a trained PGT operator: the number of TE cells biopsied, the number of pulses required to separate the biopsy from the blastocyst, the mean time needed to perform the biopsy for each operator, and the correlation to survival rates at warming [19,20]. Over the past ten years, with the progressive introduction of new diagnostic technologies, a growing necessity has emerged to re-evaluate the concept of a traditional laboratory cycle and to determine the minimum staff requirements in a lab facing PGT. Aside from the technical procedure itself, the PGT burden should also include the additional management: manually witnessing PGT workload, documentation, administrative staff related to PGT work, and an increased level of monitoring by local technical–scientific bodies [35]. Interestingly enough, Alikani and colleagues showed how the complexity of the contemporary ART laboratory is increased and requires a new look at the allocation of human resources [16]. It is of critical importance to provide laboratory directors with practical and individualized tools to determine their staffing requirements to increase the safety and efficiency of operations. As a consequence, the scenario has dramatically changed due to the introduction of extensive PGT performance, with a dramatic impact on routine workload for lab operators [36,37,38]. In addition, in the context of an IVF laboratory, structure-related key performance indicators (KPIs) are described by the type and amounts of resources in the IVF laboratory, including the staffing required to efficiently deliver a high-quality service [39]. In fact, the number of operators, staff competence, and continuous professional development (CPD) represent critical parameters for a safe and efficient workflow in a field characterized by rapidly advancing technology. An adequate number of operators is of utmost importance for an IVF laboratory, because heavy workload and insufficient personnel might lead to potential mistakes in the procedures, omissions in the manual witnessing process, or mismatches even in PGT cycles [40,41]. According to the latest guidelines for IVF laboratories, the number of operators should be set according to the maximum number of cycles, and, at minimum, two qualified operators performing all technical procedures are required [42,43]. This number should be increased according to the sum of ART procedures, as well as their complexity. In our context, the relative amounts of embryologists involved in the biopsy procedures followed the same trend as the proportion of PGT cycles to the total number of IVF cycles performed in the same study period. In addition, despite having one fewer member of staff than the smART calculator suggested, our embryology team has consistently maintained high performance and clinical outcomes. However, there is still room to further optimize the balance between operational procedures and operators involved in order to provide the highest standard of care. In particular, we demonstrated that an increase in the PGT cycles led to an increase in dedicated personnel. Starting with one embryologist trained for biopsy when PGT was first introduced in our clinic in 2015, this increased to two in 2020, one of whom was introduced as a backup to avoid service interruptions and facilitate efficient weekend rotations. The other two were introduced in 2021 and 2022, bringing the total number of embryologists dedicated to biopsies to four out of six in the lab. The fifth operator is currently under training. The training itself is a relevant factor in assessing the overall impact on laboratory workload. Training programs typically involve both theoretical components (e.g., conferences and webinars) and hands-on sessions and may range from a few to several months depending on prior expertise and the complexity of internal protocols employed. In our setting, the training period for embryologists to achieve proficiency in PGT procedures ranged from 4 to 6 months, primarily influenced by the contingent availability of degenerated embryos for practice.

We decided to train in-house embryologists in biopsy techniques because we believed that this approach would be less disruptive to our laboratory workflow and require less effort overall than recruiting an external embryologist who was already trained. The latter would have required additional time to integrate into our clinical routine. Importantly, as a similar trend was observed when comparing the number of operators involved in PGT procedures and the total number of embryologists, as well as the proportion of PGT cycles to the total IVF cycles during the study period, we suggest that an optimal balance between operational procedures and staffing levels occurs when the difference is below 10%. In addition, the total number of embryologists involved in biopsy procedures should be accurately assessed based on the highest number of blastocysts biopsied in a single working day (22 in our setting) in order to optimize the PGT-related procedures without compromising the routine IVF workflow.

Although the biopsy procedure may seem challenging, when appropriate training is performed in a standardized setting, high consistency and reproducibility of the technique can be offered with respect to both genetic and clinical outcomes [14]. The training fulfilled the recognized criteria of 50 biopsied blastocysts per operator before the training could be considered completed [19]. Moreover, the maintenance of competence level is continuously documented in a dedicated logbook to ensure long-term high-quality standards. The four embryologists involved in the biopsy showed little to no variability regarding the number of laser shots, time for biopsy and tubing, and the number of non-conclusive results, suggesting a high degree of standardization as a result of continuous monitoring of staff competence. Of note, the rate of non-conclusive results was considered a critical laboratory performance indicator, as it can be related to the embryologists’ training, as well as correct biopsy and tubing protocols [44,45,46]. In an optimal setting, the rate of non-conclusive biopsies should not exceed 5%, and our results are far below this threshold, as the number of blastocysts requiring a second biopsy accounted for 2.2% of all cycles. Euploidy rate and pregnancy rate after single euploid blastocyst transfer were not included, because they rely on clinical variables (patient characteristics and procedural protocols) that were not considered in our study. Recent studies have addressed previous concerns about the potential negative impact of long-term cryo-storage on blastocyst survival and clinical outcomes, suggesting that such effects may be less pronounced than initially feared [47,48,49,50]. Moreover, the impact of multiple biopsy and vitrification/warming cycles on reproductive outcomes following single euploid blastocyst transfer remains to be fully clarified [51,52,53,54,55,56].

## 5. Conclusions

Although based on data from a single center, our experience offers valuable guidance for other IVF laboratories in implementing PGT. It provides practical insights into training embryologists and determining appropriate staffing levels to effectively manage the additional workload. Successfully adopting best practices not only ensures high-quality patient care but also demands significant time and increased staff commitment.

## Figures and Tables

**Table 1:** Number of PGT and IVF cycles and blastocyst biopsies performed at our center during the study period (2015–2024). PGT-overall = sum of PGT-A, PGT-M+A, and PGT-SR cycles. The proportion of PGT over IVF cycles is shown.

	PGT-Overall		IVF		PGT-A			PGT-M+A			PGT-SR		
	Cycles (n)	Biopsies (n)	Cycles (n)	PGT-Overall/IVF Cycles (%)	Cycles (n)	Biopsies (n)	PGT-A/IVF Cycles (%)	Cycles (n)	Biopsies (n)	PGT-M+A/IVF Cycles (%)	Cycles (n)	Biopsies (n)	PGT-SR/IVF Cycles (%)
2015	1	3	472	0.2%	1	3	0.2%	0	0	0%	0	0	0%
2016	2	11	480	0.4%	0	0	0.0%	1	10	0.2%	1	1	0.2%
2017	5	16	468	1.1%	4	13	0.9%	0	0	0%	1	3	0.2%
2018	13	61	422	3.1%	8	33	1.9%	4	19	0.9%	1	9	0.2%
2019	68	260	391	17.4%	58	214	14.8%	7	33	1.8%	3	13	0.8%
2020	204	620	463	44.1%	191	557	41.3%	10	47	2.2%	3	16	0.6%
2021	372	1084	627	59.3%	361	1042	57.6%	8	32	1.3%	3	10	0.5%
2022	330	1063	555	59.5%	320	1008	57.7%	9	54	1.6%	1	1	0.2%
2023	338	1078	591	57.2%	330	1034	55.8%	6	26	1.0%	2	18	0.3%
2024	347	1062	476	72.9%	329	1000	69.1%	10	40	2.1%	8	22	1.7%
All	1680	5258	4945		1602	4904		55	261		23	93	

**Table 2:** Workload analysis for embryo biopsies performed. Blastocysts biopsied/cycle = the average number of blastocysts that were biopsied during each PGT cycle; range of blastocysts biopsied/patient = the minimum and maximum number of blastocysts biopsied per patient; total blastocysts biopsied/year = the total number of blastocysts that were biopsied over the one year; mean blastocysts biopsied/working day = the average number of blastocysts biopsied each day the lab is working (365 days were considered); maximum blastocysts biopsied/working day = the highest number of blastocysts biopsied in a single working day; days of biopsy/year = the total number of days in the year during which biopsies were performed.

	2015	2016	2017	2018	2019	2020	2021	2022	2023	2024
PGT cycles (n)	1	2	5	13	68	204	372	330	338	347
Blastocysts biopsied/cycle (n)	3	5.5 ± 6.4	3.2 ± 2.8	3.8 ± 2.8	3.4 ± 2.7	2.9 ± 2.2	3.0 ± 2.4	3.2 ± 2.4	3.1 ± 2.2	2.9 ± 2.0
Range of blastocysts biopsied/patient (n)	3	1–10	0–8	0–9	0–11	0–11	0–16	0–15	0–12	0–10
Total blastocysts biopsied/year (n)	3	11	16	61	260	620	1084	1063	1078	1062
Mean blastocysts biopsied/working day (n)	0.008	0.03	0.04	0.17	0.71	1.70	2.97	2.91	2.95	2.91
Maximum blastocysts biopsied/working day (n)	2	7	8	8	12	16	18	18	22	22
Working days of biopsy/year (n)	2	3	7	20	78	153	222	236	247	244

**Table 3:** Timing analysis of embryo biopsy procedures for the four embryologists trained. The years of experience, the number of biopsies performed, the number of laser shots used, the time for biopsy, the time for tubing, and the number of non-conclusive results for each embryologist are shown. The number of laser shots used, the time for biopsy, and the time for tubing are expressed as mean ± standard deviation and range. The inter-operator variability was assessed using the Kruskal–Wallis test.

Operator	Years of Experience in IVF (n)	Years of Experience in Biopsy (n)	Biopsies Performed (n)	Laser Shots (n)	Time for Biopsy (min)	Time for Tubing (min)	Inconclusive Results (%; n)
1	13	9	2092	7.5 ± 2.6 (4–12)	1.3 ± 0.5 (1–2)	1.2 ± 0.2 (1–1.8)	2.2; 46
2	8	4	2103	7.1 ± 2.1 (4–12)	1.5 ± 0.6 (1–3)	1.2 ± 0.2 (1–1.7)	2.2; 47
3	27	3	911	8.1 ± 2.9 (3–13)	1.5 ± 0.6 (1–3)	1.2 ± 0.2 (0.9–1.5)	2.3; 21
4	22	2	153	8.1 ± 2.2 (3–12)	1.6 ± 0.7 (1–3)	1.2 ± 0.3 (0.9–2)	2.0; 3
*p* value	-	-	-	0.32	0.49	0.72	0.68

**Table 4:** The current and suggested staffing levels obtained by the smART calculator [14] are shown in relation to the PGT and IVF cycles performed. The differences (Δ) between PGT and IVF operators and between IVF and the suggested level of operators are shown. The proportion of PGT staff over IVF staff, the proportion of PGT-overall cycles over IVF cycles, and the difference (Δ) between the two are also reported.

	PGT Staff (n)	IVF Staff (n)	Suggested IVF Staff (n)	Δ PGT Staff/IVF Staff (n)	Δ IVF Staff/Suggested IVF Staff (n)	PGT/IVF Staff (%)	PGT-Overall/IVF Cycles (%)	Δ Cycles (%)
2015	1	3	4.7	2	1.7	33%	0.2%	-
2016	1	4	4.9	3	0.9	25%	0.4%	-
2017	1	4	5.1	3	1.1	25%	1.1%	-
2018	1	4	5	3	1	25%	3.1%	-
2019	1	4	4.7	3	0.7	25%	17.4%	7.6%
2020	2	5	5.3	3	0.3	40%	44.1%	4.1%
2021	3	6	7.1	3	1.1	50%	59.3%	9.3%
2022	4	6	6.8	2	0.8	67%	59.5%	7.5%
2023	4	6	7.5	2	1.5	67%	57.2%	9.8%
2024	4	6	7.2	2	1.2	67%	72.9%	5.9%

## Data Availability

The data presented in this study are available on request from the corresponding author.

## Abbreviations

The following abbreviations are used in this manuscript:

PGT	Preimplantation Genetic Testing
IVF	In Vitro Fertilization
OPU	Oocyte Pick-up
DET	Double Embryo Transfer
SET	Single Embryo Transfer
PGT-M	Preimplantation Genetic Testing—Monogenic
PGT-A	Preimplantation Genetic Testing—Aneuploidies
PGT-SR	Preimplantation Genetic Testing—Structural Rearrangements
CCT	Comprehensive Chromosome Testing
CGT	Carrier Genetic Testing
AFC	Antral Follicle Count
AMH	Anti-Mullerian Hormone
ICM	Inner Cell Mass
TE	Trophectoderm
NGS	Next-Generation Sequencing
